# Meeting of the American Medical Association

**Published:** 1850-06

**Authors:** 


### EDITORIAL DEPARTMENT.

*Meeting of the American Medical Association.*—The third annual meeting of the *American Medical Association* was held at Cincinnati on the 7th. ultimo. The session continued four days, about three hundred and fifty delegates and members (by estimation) being in attendance, representing twenty-four states of the Union. The interesting character of the proceedings, will not, we are sure, suffer, in the minds of those present, by comparison with any of the previous meetings. We are not prepared to present our readers with a complete report of the proceedings. The accounts published in the daily papers are too meagre and inaccurate to be available, and we must therefore await the publication of the volume of Transactions, which, it is to be hoped, will appear more promptly than the volume for the last year. It will also obviously be more appropriate to defer notices of the reports of the standing committees, (some of which were very able,) until they are in print. We shall confine ourselves, now, to a brief sketch of the doings of the Association.

After an address by the late President, John C. Warren, M. D., of Boston, Mass., on taking the chair, and appointing a Committee on the claims of those proposed as "members by invitation," the Association proceeded to the business of choosing officers for the ensuing year. The mode of nominating, is by a committee consisting of one member from each state represented, the member for each state being appointed by the delegation from that state. The following officers were nominated, and elected :—

President, R. D. Mussey, M. D. of Ohio.
1st. Vice President, J. B. Johnson, M. D., of Missouri.
2d. do. A. Lopez, M. D., of Alabama.
3d. do. Daniel Brainard, M. D., of Illinois.
4th. do. G. W. Norris, M. D., of Pennsylvania.
Secretaries, { A. Stille, M. D., of Pennsylvania.
N. Desaussure, M. D., of South Carolina. }
Treasurer, Isaac Hays, M. D., of Pennsylvania.

A resolution embodying a tribute of respect to the memory of the late Prof. Harrison, of Cincinnati, one of the Vice Presidents chosen at the last meeting of the Association, was adopted in an impressive manner, the members signifying their assent by rising.

The newly elected officers were then inducted into office, the retiring President making a short address, which was followed by a few remarks by Dr. Mussey, on taking the Chair. A vote of thanks to the late officers was then passed.

Considerable discussion arose on the propriety of adopting more restrictive measures than the Constitution at present provides for, respecting the reception of members by invitation. None of the plans proposed, however, appearing to meet the favor of a majority of the body, the subject, at length, was indefinitely postponed.

The report on Medical Education by the chairman of the committee on this subject, Joseph Roby, M. D., of Maryland, was the first of the reports of the standing committees read. After the reading of the report, Dr. Blatchford, of N. Y., a member of the committee, submitted a series of resolutions, embracing recommendations with respect to extension of the terms of lectures in Medical schools, and clinical instruction ; not contained in the report presented by the chairman. The questian on the adoption of these resolutions occasioned much and animated debate, which was not terminated until noon of the third day of the session Among those who engaged in discussions connected with this subject, were Drs. Miller and D. W. Yandell of Louisville, Ky., Annan of Lexington, Ky., J. K. Mitchell, Caspar Morris and Isaac Parrish of Philadelphia, Brainard and Davis of Chicago, Ill., Kerfoot, of Lancaster, Pa., Ware and Storer, of Boston, Mass., Wright and Rieves, of Cincinnati, O., McPheters, of St. Louis, Mo.

The subject of Medical education not only consumed more time, but it involved more exciting topics than any other of the subjects considered during the session. It was finally disposed of by referring the Report of Dr. Roby to the committee on Publication, and the Resolutions of Dr. Blatchford to the committee on Education appointed to report at the next meeting of the Association.

This result suffices to show (what must have been apparent to the majority of the members present) the inutility of devoting so large a portion of the session to deliberations on proposed improvements in Medical instruction. That the subject is one of great importance, and deserves a proportionate share of the attention of the Association, no one will deny ; but it is not to be forgotten that there are other subjects which should neither be passed by, nor slighted. It is but one of the objects of the Association to legislate in behalf of those who are entering, or hereafter to enter the medical profession. To develope and diffuse information among the members of the Association, and their professional brethren, is another object scarcely less important. And for this end, time should be allowed for the reading of all reports, and voluntary communications, on the various branches of medical science, and for discussions therewith connected. Numerous topics also, in addition to those just referred to, claim consideration, in which the honor and usefulness of the Medical profession are more or less involved. At each meeting of the Association, hitherto, the subject of Medical Education has absorbed so much time, that other matters have received less attention than justly belongs to them. We trust there may be an improvement in this particular hereafter, more especially since the discrepancy of opinion as regards medical instruction, appears to increase in proportion to the amount of discussion it receives. Under existing circumstances, it seems to us that the various questions relating to this imporiant subject had better be argued at length in the different Medical journals, and in local associations, awarding them only a fair relative degree of consideration at the meetings of the Association.

The Report of the Committee on Practical Medicine was next read by the Chairman, Prof. J. K. Mitchell, of Philadelphia. The reading of this Report occupied about two hours. It was chiefly devoted to Epidemic Cholera.

The Report of Prof. Huston, (Chairman) on the Adulteration of Medicines, was next read.

The Report on Surgery followed, Prof, R. D. Mussey, Chairman. This occupied about two hours. Much of this report was made extemporaneously.

Dr. Stille, Chairman of Committee on Medical Literature, submitted his Report, an able and elegant production.

The Report on the Medical Sciences, Dr. Parsons, of Providence, R, I., Chairman, owing to the ill-health of Dr. P., was not read, but referred to the Committee on Publication.

The Commitee on Obstetrics had not prepared a Report. Prof. Evans, of Chicago; Ill., (a member of the Committee) exhibited and explained a new instrument, invented by himself, designed as a substitute for the obstetrical forceps, of which we shall give some account in another article.

Several voluntary communications were presented, and referred to the committee on Publication, without reading. A paper by Prof. Davis, of Chicago, on the relation of the cerebellum to the sexual propensity, was read.

As already stated, we deem it more appropriate to defer analyses or critical notices of the reports and communications presented, until the appearance of the volume of Transactions.

Various resolutions were adopted during the session, on which, not having copies, we must defer comment, until an authentic publication of the proceedings is received. Among the objects embraced in these resolutions are the following ;

Recommendation to Congress of the application of Navy Surgeons for an assimilated rank in the service ; the publication by the Association of the manuscript work of the late Dr, Forrey ; memorial to Congress for laws regulating international copy-rights ; advising the introduction of the practical study of chemistry into medical schools ; appointing a committee to report on the diseases of animals ; showing the importance of encouraging native medical literature, by patronizing American text-books; recommendation to the Profession to restrict their patronage to apothecaries not engaged in making or vending secret nostrums, and to repudiate members of the profession who give certificates of the value of patent remedies ; approving of clinical midwifery, and referring the subject to the committee on Education for the coming year. Presuming that some of our readers might feel interested in the last-mentioned topic, from the communications and articles thereupon that have appeared in our pages, we obtained an attested copy of the following preamble and resolution, which were offered by Prof. Miller, of Louisville, Ky., and adopted by an *unanimous* vote of the Association :

"Whereas clinical instruction in Medicine and Surgery is now generally acknowledged to be essential to the proper qualification of students for the practice of these branches of our profession, and whereas it must be admitted that clinical instruction in midwifery would be equally valuable—

" *Resolved*, That the committee on Medical Education be instructed to inquire whether any practicable scheme can be devised to render instruction in Midwifery more practical than it has hitherto been in the Medical Schools of the United States, and report at the next meeting of this Association."

The following are the standing committees for the present year :

*Medical Science.*—Dr. Bennett Dowler, of New Orleans, La., Chairman; Drs. Fenner, of New Orleans, T. G. Smith, of Philadelphia, Uhsher, Va, Carr, N. Y., Johnson, Ala., Meeks, Indiana.

*Practical Medicine.*—Dr. Austin Flint, of Buffalo, N. Y., Chairman ; Drs. N. L. Corbin, Va., R. H. Davis, Baltimore, Md., H. M. Congar, Buffalo, N. Y., J. McNaughton, Albany, N. Y., Norwood, New Orleans, R. Haywood, Indiana.

*Surgery.*—Dr. Paul F. Eve, Augusta, Ga., Chairman ; Drs. J. N. Simmons, Ga., Gross, Ky., J. Watson, N. Y., A. Pope, St. Louis, Mo., H. H. McGuire, Winchester, Va., Palmer, Michigan.

*Obstetrics.*—Dr. Storer of Boston, Mass., Chairman ; Drs. Reynolds Boston, Mass., T. M. Smith, Delaware, Miller, Louisville, Ky., Parker, Wisconsin, Morrison, Illinois, Morton, Indiana.

*Medical Education.*—Dr. Worthington Hooker, Norwich, Conn., Chairman ; Drs. Blatchford, Troy, N. Y., J. B. S. Jackson, Boston, Mass., N. S. Davis, Chicago, Ill., Theobald, Baltimore, Blackburn, Ky.

*Medical Literature.*—Dr. Thomas Reyburn, of St. Louis, Mo., Chairman ; Drs. McPheters, St. Louis, Mo., Cowper, Newcastle, Del., Lawson, Cincinnati, Ohio, G. Tyler, D. C., Annan, Lexington, Ky., Thomas, Tenn.

*On Publication.*—Dr. Isaac Hays, of Philadelphia, Chairman : Drs Alfred Stille, Philadelphia, Dunbar, Baltimore, D, F. Condie, Philadelphia Isaac Parrish, Philadelphia, S. DeSausure, Charleston, S. C., Sanborn. N. H

The city of *Charleston*, S; C., was designated as the place of meeting, on the first Tuesday in May, 1851, and the following committee of Arrangements appointed :

Dr. Frost, Charleston, S. C., Chairman ; Drs. P. C. Gaillard, Charleston, S. C., J. P. Jervey, Do., DeSaussure, Do., Jebby, Do., Wragg, Do., Cain, Do.

The above enumeration of members of the committees is copied from a report in one of the daily newspapers of Cincinnati, and may contain errors, which, when ascertained, we shall correct.

As before stated, the late Association at Cincinnati will compare favorably as respects the interesting character of its proceedings, with any of the previous meetings . The harmony and good feeling that have characterized the deliberations hitherto, were not less conspicuously displayed on this occasion. Differences of opinion were freely expressed, but with due regard to propriety and gentlemanly courtesy ; and although the tone of discussion was often far from being tame and spiritless, it was generally free from personalities and asperity. Members were not disposed to extend the licence of debate to the latitude of licentiousness ; and hence, the violations of strict order and decorum were few in number. The measures adopted by the Association as a legislative body, show a temperate, conservative disposition. The scientific reports are of such a character that the volume of Transactions for this year will continue to be a fair exponent of the progress of American medicine. And, altogether, the claims of the Association upon the deep interest and fostering care of the Medical Profession of this country, will be found to be in no wise diminished.

But the advantages of these annual occasions for assembling medical practitioners from all parts of the United States, are by no means limited to the formal proceedings, and the published reports. The social intercourse of the members with each other, and with their medical brethren in the place of meeting and its neighborhood; the formation and renewal of friendships, and the opportunity of seeing, hearing, and knowing many with whose names and writings we were already familiar, render these occasions as delightful as they are improving. We were at some pains to ascertain the sentiments of our brethren who had come from distant States to attend the meeting at Cincinnati, and we found none who did not express themselves more than abundantly repaid for the loss of time, and the expense, and the fatigues, which their attendance required, by the gratification derived from the sources just mentioned.

The local popular influence exerted by these meetings in the places at which they are held, is to be included among their beneficial results. To witness such a body convened for objects of science and philanthropy, cannot but impress the minds of citizens in a favorable manner, and must tend to elevate the medical profession in the respect and estimation of the community. On this account, it is desirable that the annual meetings shall be held on each year at different sections of the country, as the Constitution provides; and it is not to be doubted that the moral effect produced in all places in which they are held, render it a matter of policy, as well as pride, for different places to compete for the honor of being selected. The position of Buffalo is such, that after the next annual meeting at Charleston, S. C., it may present strong claims for preference, and we hope that our brethren in Western New York will not fail duly to appreciate the importance of so desirable an event.

In closing this imperfect sketch, it remains to allude to the judicious and liberal arrangements, the social attentions, and the generous hospitalities of our brethren of Cincinnati. To be fully appreciated, these must have been experienced. To those who were present, it were needless to attempt to express sentiments of appreciation, for description could not add to what is already felt by all. Were we able to do justice to this theme, it would tend to enhance in no small degree, the sense of disappointment of those who were unable to be present. To the latter, therefore, we will simply tender our condolence. They who were privileged to participate in the Session of 1850, will ever preserve happy and grateful recollections of the chairman of the committee of arrangements, (Dr. Drake,) and his compeers, in the Queen city of the West.

